# Is COVID-19 perceived as a threat to equal career opportunities amongst Swiss medical students? A cross-sectional survey study from Bern and Geneva

**DOI:** 10.3205/zma001586

**Published:** 2023-02-15

**Authors:** Benny Wohlfarth, Meghan M. McConnell, Michaël Huguenin-Dezot, Mathieu Nendaz, Reto M. Kaderli

**Affiliations:** 1 University of Bern, Bern University Hospital, Department of Visceral Surgery and Medicine, Bern, Switzerland; 2 University of Ottawa, Department of Innovation in Medical Education, Ottawa, Canada; 3 University of Ottawa, Department of Anesthesiology and Pain Medicine, Ottawa, Canada; 4 University of Geneva, Department of Development and Research in Medical Education, Geneva, Switzerland

**Keywords:** medical students; pandemic, loss of part-time jobs, living expenses, equal career opportunities, socioeconomic status

## Abstract

**Introduction:**

Students frequently rely on part-time jobs to earn a living wage. We sought to evaluate the sociodemographic status of Swiss medical students and their perception regarding equal career opportunities in view of impaired part-time job opportunities under the COVID-19 pandemic.

**Methods:**

We conducted an anonymous online survey among Swiss medical students from Bern and Geneva over a period of 4 months between December 2020 and April 2021. We evaluated sociodemographic data, current living situation, part-time job occupation as well as other sources of income to fund living expenses, and, by means of a five-point Likert scale (1=strongly disagree and 5=strongly agree), whether COVID-19 was perceived as impeding equal career opportunities.

**Results:**

Of 968 participants, corresponding to around 13.8% of all medical students in Switzerland, 81.3% had part-time jobs. Amongst the employed, 54.8% worked to afford living expenses and 28.9% reported a negative financial impact due to reduced part-time jobs under the pandemic. The loss of part-time jobs was perceived to make medical studies a privilege for students with higher socioeconomic status (4.11±1.0), whose opportunity to study is independent of a regular income. A governmental backup plan was considered crucial to support affected students (4.22±0.91).

**Discussion:**

COVID-19 and its sequelae are perceived as a threat for Swiss medical students and lead to a disadvantage for those with lower socioeconomic status. Nationwide measures should be established to foster equal career opportunities.

## Zusammenfassung

**Einleitung:**

Studierende sind häufig auf Nebenjobs angewiesen, um ihren Lebensunterhalt zu bestreiten. Wir untersuchten den soziodemographischen Status von Schweizer Medizinstudierenden und deren Einschätzung, ob reduzierte Teilzeitarbeitsmöglichkeiten im Rahmen der COVID-19-Pandemie eine Bedrohung der Chancengleichheit zur Erlangung des Berufes darstellt.

**Methoden:**

Wir unternahmen eine anonyme Online-Umfrage unter Schweizer Medizinstudierenden aus Bern und Genf über einen Zeitraum von 4 Monaten zwischen Dezember 2020 und April 2021. Abgefragt wurden soziodemografische Daten, die aktuelle Lebenssituation, Teilzeitbeschäftigung sowie etwaige weitere Einkommensquellen zur Finanzierung des Lebensunterhalts. Zudem wurde mittels einer fünfstufigen Likert-Skala (1=stimmt überhaupt nicht zu, 5=stimmt voll und ganz zu) abgefragt, ob die Auswirkung der COVID-19-Pandemie hierauf als Beeinträchtigung der beruflichen Chancengleichheit wahrgenommen wird.

**Ergebnisse:**

Von den 968 Teilnehmenden, die rund 13,8% aller Medizinstudierenden in der Schweiz darstellen, hatten 81,3% einen Teilzeitjob. Von den Erwerbstätigen arbeiteten 54,8% neben dem Studium, um den Lebensunterhalt zu bestreiten, wobei 28,9 % über negative finanzielle Auswirkungen aufgrund der reduzierten Teilzeitstellen während der Pandemie berichteten. Der Verlust von Teilzeitjobs machte das Medizinstudium aus Sicht der Studierenden zu einem Privileg für Studierende mit höherem sozioökonomischem Status (4,11±1,0), deren Möglichkeit zu studieren eher unabhängig von einem regelmäßigen Einkommen ist. Ein staatlicher Notfallplan für betroffene Studierende wurde dabei als wichtige Massnahme zur Unterstützung angesehen (4,22±0,91).

**Diskussion:**

Die COVID-19-Pandemie und ihre Folgen werden von den Befragten als Bedrohung wahrgenommen und bringen eine Benachteiligung für Schweizer Medizinstudierende mit niedrigerem sozioökonomischem Status. Es sollten gesamtschweizerische Massnahmen zur Förderung der beruflichen Chancengleichheit ergriffen werden.

## Introduction

The corona virus disease 2019 (COVID-19) pandemic has affected almost every aspect of daily life [1], [2]. As a result of the long incubation period and high transmission rate [3], governments implemented drastic measures to lessen the spread of the virus, including temporary public lockdowns, workplace closures, restrictions on domestic and international travel, and closing of educational institutions [4]. Such restrictions have affected various industries, businesses, and organizations worldwide, resulting in job losses and reduced working hours [5], [6], [7].

The COVID-19 pandemic has also had a substantial impact on educational practices, for example transitioning to online learning platforms, introducing new assessment methods, and cancelling elective training opportunities [8], [9]. Within higher education, many students report financial concerns and hardships resulting from COVID-19. For example, a survey among approximately 1500 undergraduate students found that working students experienced a 31% decrease in their wages and a 37% drop in their weekly working hours and for 13% COVID-19 led to a delay in graduation [10]. Moreover, approximately 40% of surveyed students reported that they lost a job, internship, or job offer. Similarly, it was found that over 80% of college students reported that they and/or someone in their household lost income as a result of the pandemic [11]. Such consequences are concerning, as many students rely on part-time jobs to cover living and leisure expenses, gain working experience and accomplish independency [12], [13]. As mentioned above, several studies have documented the impact of COVID-19 within higher education in general [10], [11], [14]. Given the fact that Switzerland is one of the most expensive countries in the world [15], a better understanding of the pandemic’s impact on medical students seems crucial. To our knowledge, no research to date has examined whether COVID-19 is a potential threat for equal career opportunities amongst Swiss medical students. Therefore, the purpose of this study was to evaluate sociodemographic data, current living situation, part-time job occupation and other sources of income to fund living expenses, and whether COVID-19 was perceived as impeding equal career opportunities.

## Methods

### Study design

We conducted a cross-sectional survey study at two Swiss Universities: University of Bern, and University of Geneva. The Ethics Commission of the Canton Bern, Switzerland determined that ethical approval was not required for this study (BASEC-No: Req-2020-00662).

#### Survey instrument

The electronic survey was written in both French and German, and consisted of 33 items (see attachment 1 ). The survey was designed and programmed by the authors using Qualtrics^®^ Core XM Survey Software (Qualtrics, Provo, Utah). Invitation links were sent out via e-mail to all medical students from the Universities of Bern and Geneva in mid-December 2020. Response enhancement techniques included advanced notification. Two reminder emails were circulated at the beginning and middle of January 2021. The survey remained open until mid-April 2021 to give students, who might have been busy working in the winter semester break, the chance to participate.

The survey consisted of three sections. The first section contained 10 closed-ended items related to students’ demographic characteristics (e.g., age, gender, year started medical school training) and family background (e.g., parental education and occupation). The second section was based on 13 items and collected data on students’ finances. For example, students were asked to identify their current living situation, the nature of their financial support, and whether they held a part-time job. Students who indicated they had part-time employment were further asked to describe the nature of their employment, the number of working hours, and their reasons for working. The third section of the survey consisted of 10 items that asked students to rate their perceptions regarding the financial impact of COVID-19. Five of these items assessed the impact of COVID-19 on their personal finances, and the other five items evaluated the impact of COVID-19 on medical students in general. Each item was rated on a five-point Likert scale, scored by 1=strongly disagree and 5=strongly agree.

#### Statistical analysis

We used descriptive statistics, including measures of central tendency (mean, median), dispersion for continuous variables (standard deviation), and counts and percentages for categorical data. To explore subgroup differences among participants with part-time employment, we used independent t-tests and chi-square tests for continuous and categorical data, respectively. For data analysis, we used SPSS version 27.0 (IBM, Armonk, NY).

## Results

### Participant characteristics

We collected data from 968 medical students (738 from Bern and 230 from Geneva, corresponding to a response rate of 43.2% and 17.9%, respectively), which account for 13.8% of all medical students in Switzerland [16]. Demographic characteristics are presented in table 1 (Tab. 1). The mean age of participants was 22.4±2.0 years, and the most of respondents were female (64.4%). The majority of respondents (83.6%) started medical school between 2016 and 2020. Similarly, most respondents reported living with either family members or roommates (86.3%), with only 11.8% living on their own. For those respondents who reported having to pay rent/mortgage, the average cost was CHF 980±717 per month.

With regards to family background, the majority of respondents reported that their parents had some form of postsecondary education (79.5%) and worked in either skilled or professional occupations (91.6%). Nearly half of respondents (47.9%) reported that their parents held managerial or executive positions.

Respondents were asked to identify sources of financial support. The majority of the respondents reported receiving financial support from their parents and family members (64.8%). Nearly half of respondents also identified personal savings as a source of financial support (45.1%). Only 10.8% of respondents relied on scholarships or loans.

#### Part-time job characteristics

Overall, 81.3% of the respondents reported having a part-time job. Respondents with part-time employment were significantly older than those without part-time jobs (22.9±2.8 years vs. 20.7±1.9 years, p<0.001). Correspondingly, part-time job status was associated with the year of starting medical school training (χ^2^=167.5, p<0.001).

Part-time job status and current living situation were significantly associated (χ^2^=25.1, p<0.001): analysis of subgroup differences showed that 87.0% of respondents living on their own and 87.4% living with roommates had part-time jobs. Amongst respondents that live with family members, only 74.3% were working part-time besides their medical training. Furthermore, respondents with part-time jobs reported significantly lower costs of rent/mortgage than those without part-time jobs (CHF 1,305±958 vs. CHF 919±643, p<0.001). Part-time job status was not associated with parental education (p=0.14) and parental occupation (p=0.38), respectively. 

Table 2 (Tab. 2) shows the job characteristics of respondents working part-time during medical school. The majority (77.5%) of respondents reported that the purpose of their part-time employment was to afford leisure expenses, while nearly half of the respondents reported working part-time to afford living expenses (54.8%) and/or gain work experience (49.8%). Overall, 71.7% of respondents had a part-time job in a hospital or non-hospital healthcare setting, followed by restaurants (17.7%) and private tutoring (17.1%). For 20.0% of respondents, their employment setting was not among the listed options and their write-in responses included non-private tutoring, sport and social work. Almost half of participants (47.6%) had more than one employer and therefore more than one part-time job. Lastly, 28.9% of respondents reported that their finances were impacted negatively by COVID-19 as a result of job losses or reduced working hours.

#### Perceptions of the economic impact of COVID-19

Respondents’ ratings of the negative impact of COVID-19 on their personal financial situation and on medical students in general are shown in table 3 (Tab. 3). Overall, respondents perceived COVID-19 to have a more negative impact on the finances of medical students in general compared to themselves (2.0±0.99 vs. 4.0±0.63, p<.001). Respondents perceived students with a lower socioeconomic status to have to work harder in order to stay in medical school (4.31±0.97). Respondents believed that the loss of part-time jobs to afford medical school leads to a privilege for students with a higher SES (4.11±1.0). Furthermore, the participants stated that the government should have a backup plan, which allows students in need to stay in medical school (4.22±0.91).

## Discussion

Students frequently rely on part-time jobs to earn a living wage. In Switzerland, as one of the most expensive countries in the world [15], medical students find themselves exposed to high living costs, especially with regards to housing and daily expenditures, while pursuing a graduation in one of the longest and most time consuming fields of study.

To the best of our knowledge, we are the first to evaluate whether COVID-19 is a potential threat for equal career opportunities amongst Swiss medical students due to job loss or reduced working hours. In the present study, more than three quarters of medical students reported having at least one part-time job, with half of them stating that their reason for working was to afford living expenses. Nearly one-third reported a negative effect of the COVID-19 pandemic on their finances.

Reliance on part-time employment by medical students differs between countries [17], [18], [19]. In Switzerland, the percentage of medical students with part-time jobs is lower than in other fields of tertiary education according to the latest reports from the Federal Statistical Office [20], [21]. Based on these reports, one possible reason for this discrepancy may be the time consuming nature of medical training due to the large number of mandatory courses and internships. The latter reduce the time available for part-time employment compared to other fields of study in Switzerland [20], [21].

Consequently, we found that many participants rely at least partly on financial support from their parents or relatives. Yet, nearly half of medical students reported having established financial reserves through personal savings. As the autonomous generation of such savings requires income, part-time jobs play a pivotal role in this form of financial backup. 

Almost half of the participants from our study hold more than one part-time job, and one-fifth works more than 30 hours per month. However, almost one third of participants had already experienced job loss or reduced work hours due to COVID-19. This finding is in line with reports from other countries' medical students [22] and other more general studies from the higher education sector [10]. Considering the cost of living in Switzerland, the reduction of part-time job opportunities is a fundamental problem, especially for the 35% of medical students who reported an absence of financial support from their parents or relatives. Accordingly, the majority of respondents’ perceived the impact of the pandemic as a disadvantage for medical students with a low SES. According to them, medical students with a lower SES have to work harder to stay in medical school and the loss of part-time job opportunities leads to a privilege for students with a higher SES, whose opportunity to study is independent of a regular income. Differences in the SES are therefore perceived as a threat to equal career opportunities. 

The potential benefits of securing and increasing the representation of medical students from lower SES backgrounds have been well described in the literature. Besides creating social justice and mobility [23], these graduates are more likely to work in primary care and serve communities with similar backgrounds and characteristics [24], [25]. Considering the increasing shortage of doctors especially in the primary care sector [26], [27], the integration and successful graduation of medical students with lower SES appears to be a sensible measure towards improving the primary care situation in Switzerland.

Prospectively, the medical students in our study expect a further negative trend of the overall economic situation in the next months and even years due to COVID-19. Consequently, the majority of participants would support a backup plan from the government for medical students experiencing financial difficulties in order to counteract the disadvantage of students with a lower SES. 

The temporary increase of COVID-19 associated part-time jobs in the healthcare sector (e.g., working in test centers, wards or on intensive care wards), may represent an appropriate employment opportunity for some but not all students that rely on part-time jobs to compensate for the overall loss of working hours from other industries. 

There are several emergency funds from universities and foundations in Switzerland (e.g., EDUCA SWISS), with some of them offering support without repayment obligations. We are not aware as to what extent Swiss medical students are informed of such funds, but in view of the overwhelming number of vulnerable students, these emergency funds should be supported and appropriately mediated by a nationwide concerted approach to secure equal career opportunities and diversity in the future workforce of medicine.

In contrast to other western countries (e.g. Canada or the United States) [28], [29], external financial backups in the form of scholarships or loans are not widely available to Swiss medical students. Scholarships are considered targeted subsidies in Switzerland, where regulations and figures may vary significantly between cantons [30]. An initiative to harmonize these regulations on a national level has been declined in 2015 [31]. Finally, traditional scholarships may not qualify as a viable option for emergency scenarios because of their significant lead-time.

The Swiss Conference of Cantonal Ministers of Education advised that indebtedness of young people should not be considered a mainstay in the social and educational policies of Switzerland [30]. Especially for students with a lower SES, repayment and loan securities may present obstacles considering their lower collateral and the higher likelihood to withdraw from medical school before graduation [32]. Ultimately, this could leave these students indebted without a sufficient chance for repayment, causing hardship cases and high social burden [30], [33].

We found that the reasons to take on a part-time job were not only to afford living and leisure expenses, but also to gain working experience in the healthcare sector, which is in accordance with previous recommendations and findings [19], [34]. Part-time jobs give students the opportunity to gain experience in various specialties, which is important for developing a more differentiated decision regarding future career choices [35]. Therefore, the current reduction in non-COVID-19 related part-time job opportunities also holds a risk for specialties that already have recruitment difficulties.

More generally, the early to mid-twenties are also a crucial period in the transition to adulthood, where part-time jobs play an important role for independency and personality formation [36]. Accordingly, we found that Swiss medical students with part-time jobs were less likely to live with family members and were older, which is in line with earlier findings [37]. By including medical students from the Universities of Bern and Geneva, we covered both linguistic regions for medical studies in Switzerland. However, a major limitation of the study is its geographically limited sample of Swiss medical students, which does not enable generalization to other areas. Nevertheless, similar situations may occur in other western countries where medical students have limited access to external financial backups. Given the offset between an external event such as a global pandemic and economic effects, the main impact on equal career opportunities for medical students may still lie ahead. Our study is also susceptible to sampling bias due to a low response rate in Geneva and as medical students who were particularly impacted by COVID-19 may have been more likely to respond. Invitation links were sent out in the winter semester break, which may have led to an underrepresentation of medical students with a lower SES due to their work obligations. To counteract this effect, we kept the survey open up until mid-April. Lastly, we only asked students whether they worked part-time while in medical school, but not whether they worked continuously, seasonally or casually. However, a casual employment seems unlikely with regard to the reported amount of working hours. 

In conclusion, our data underline the negative impact of the economic aftermath of the COVID-19 pandemic on equal career opportunities for many Swiss medical students, especially for those with a lower SES. To achieve equal career opportunities independent of the SES, a nationwide measure should be established to provide financial support when necessary.

## Declarations

### Ethics approval and consent to participate

- Ethics approval not needed as ruled by the Swiss ethics committee in the Cantone of Bern (BASEC-No: Req-2020-00662), consent was implicitly given by the participant by submission of the survey.
- We confirm that all methods were carried out in accordance with relevant guidelines and regulations.

#### Availability of data 

Data are available from the corresponding author upon request.

#### Authors' contributions

- BW: PI, concept, literature search, writer of the manuscript
- MM: Statistician, literature search, critical review of the manuscript
- MH: Concept, French translation of survey, email and reminders
- MN: Local PI Geneva, critical review of the manuscript
- RK: Concept, critical review of the manuscript

All authors read and approved the final manuscript.

## Acknowledgements

We are grateful to Peter Frey, MD, Head of Unit of Student’s Affairs, Faculty of Medicine, University of Bern for the support in distributing the electronic survey among the medical students at the University of Bern.

We are also grateful to David Köckerling, MD, Department of Angiology, Bern University Hospital, University of Bern, Bern, Switzerland for language editing and proofreading of the manuscript.

## Competing interests

The authors declare that they have no competing interests. 

## Supplementary Material

Survey

## Figures and Tables

**Table 1:**
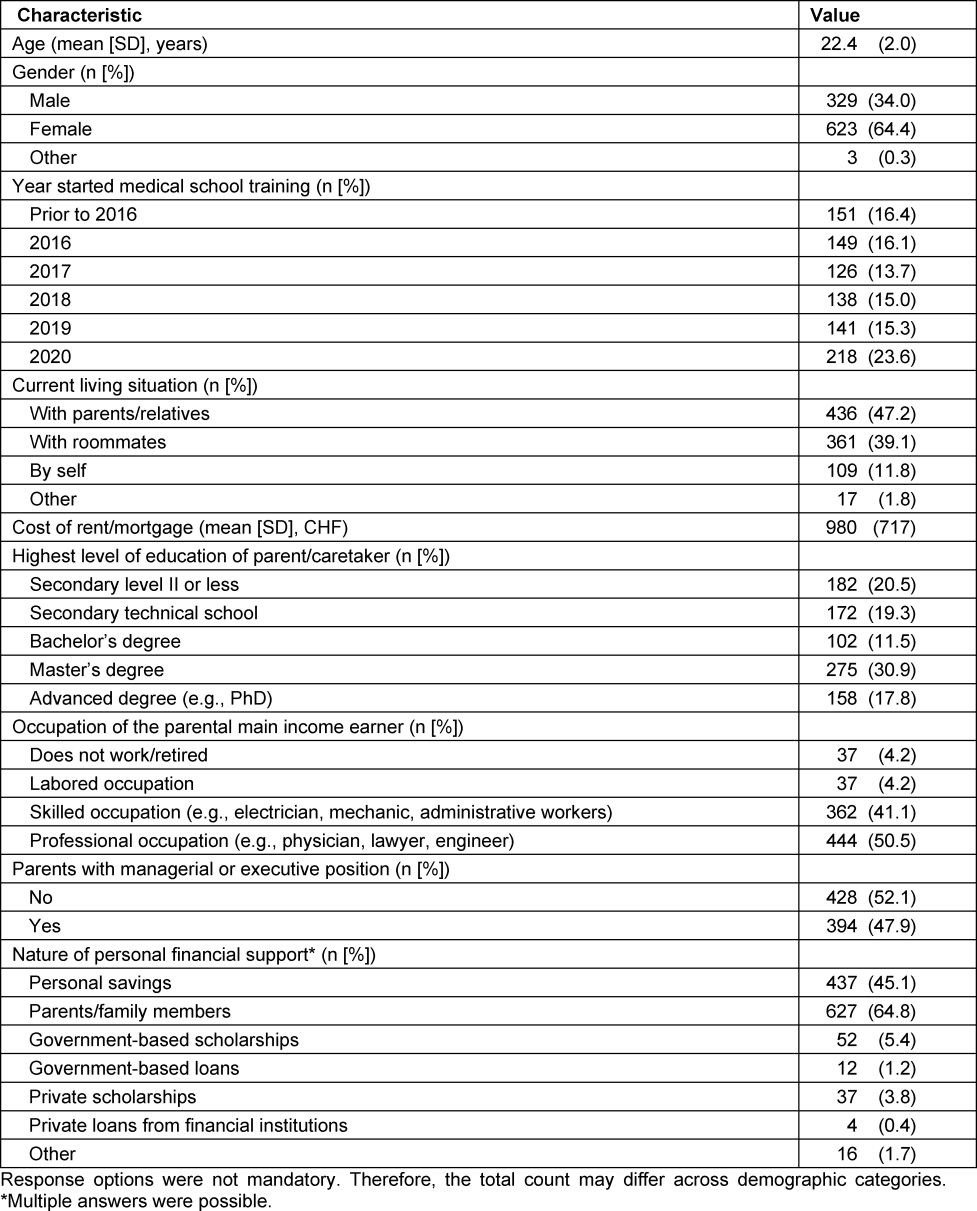
Respondents’ characteristics

**Table 2:**
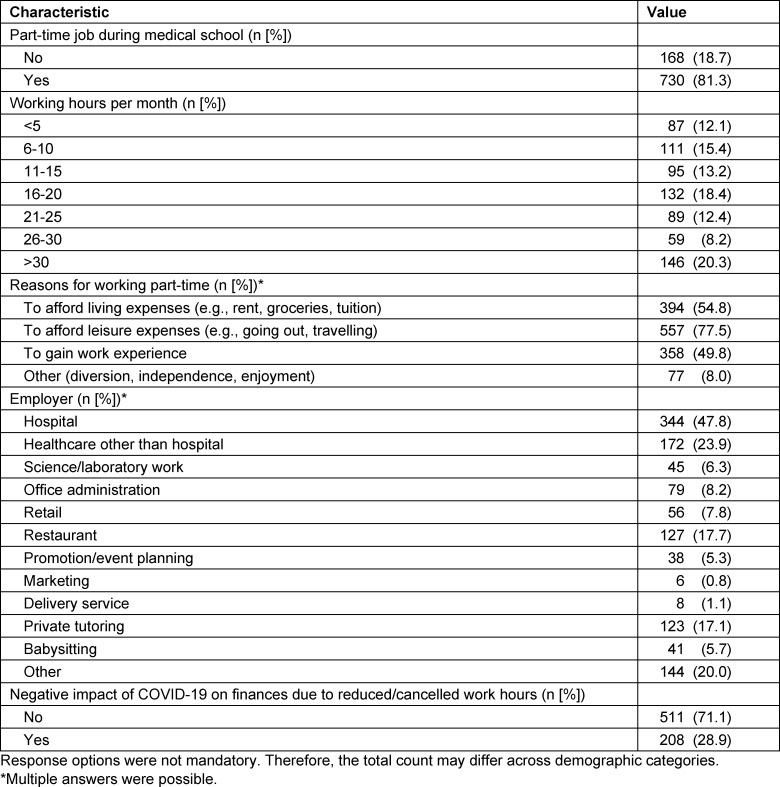
Job characteristics of medical students working part-time during medical school

**Table 3:**
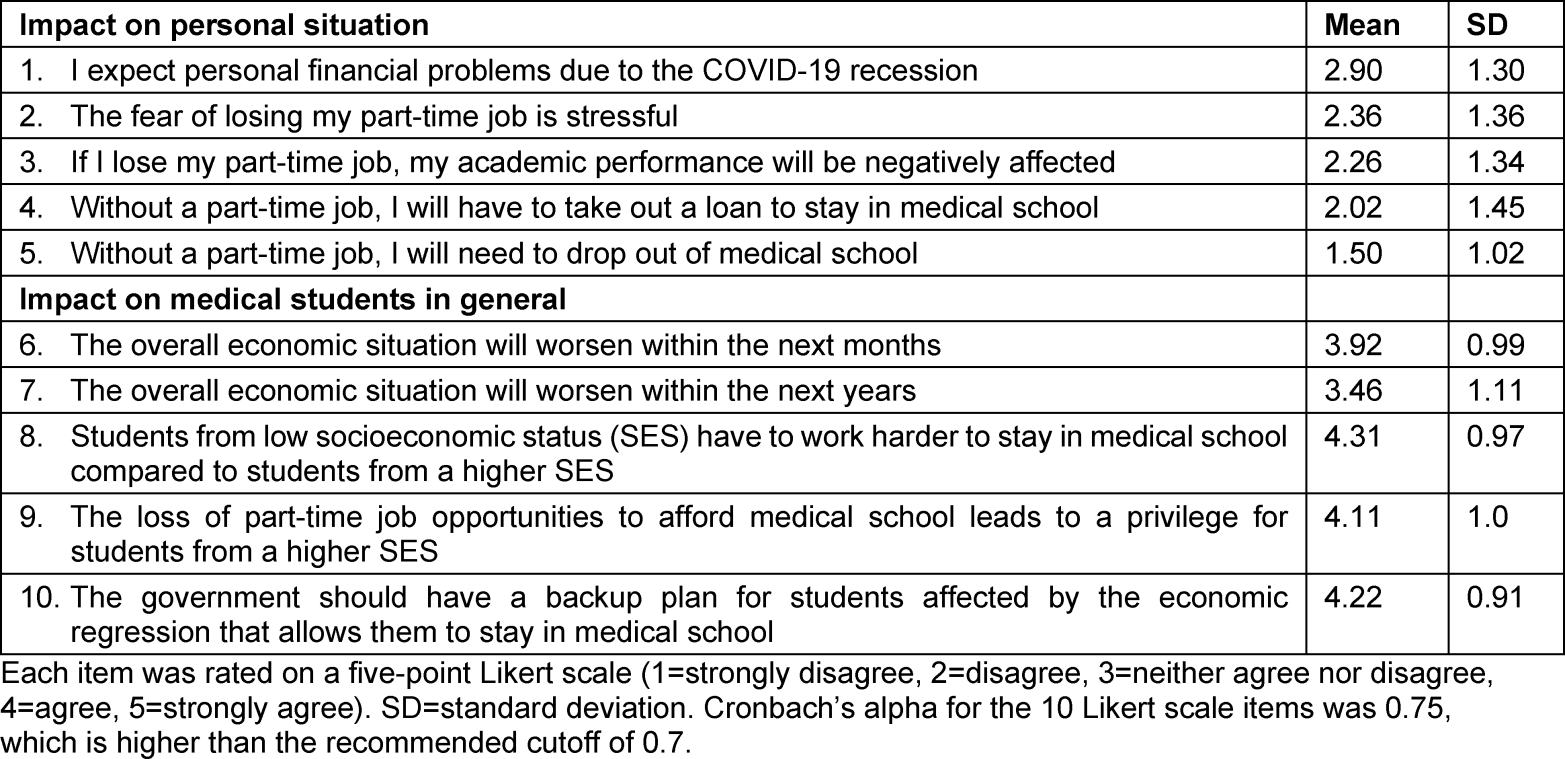
Perception of the financial impact of COVID-19 on the personal situation and on medical students in general
