# Supplementary material for: Is COVID-19 perceived as a threat to equal career opportunities amongst Swiss medical students? A cross-sectional survey study from Bern and Geneva
Source: GMS J Med Educ. 2023 Feb 15;40(1):Doc4. doi: 10.3205/zma001586 (PMC10010764; doi:10.3205/zma001586)
Supplement: Survey [file JME-40-4-s-001.pdf]

Attachment 1: Survey

What is your age?

____

What is your gender?

- O Male
- O Female
- O Non-binary
- O I do not want to answer

Are you a permanent resident of Switzerland?

- O Yes
- O No

If not, did you come to Switzerland specifically to attend medical school?

- O Yes
- O No

What year did you start your medical school training?

____

What is your current living situation?

- O I live with my parents
- O I live with relatives (other than my parents)
- O I rent an apartment/house with roommates
- O I rent an apartment/house by myself
- O I live in an apartment/house that I own
- O Other (please specify) ____

How much is your monthly rent/mortgage in Swiss francs (CHF)?

____

What is the highest level of education your parents/caretakers have completed?

- O No graduation
- O Less than High School
- O High School
- O College
- O Bachelor's degree
- O Master's degree
- O Advanced degree, e.g., PhD, MD (please specify)
____
- O Unknown / not applicable

Attachment 1 to Wohlfarth B, McConnell MM, Huguenin-Dezot M, Nendaz M, Kaderli RM. *Is COVID-19 perceived as a threat to equal career opportunities amongst Swiss medical students? A cross-sectional survey study from Bern and Geneva.* GMS J Med Educ. 2023;40(1):Doc4. DOI: 10.3205/zma001586

Between your parents, what is the occupation of the main income earner?

O No occupation

O Labored occupation (e.g. parking lot attendant, janitor, information desk clerk, etc.)

O Skilled occupation (e.g. electrician, mechanic, administrative workers, insurance and other financial clerks, etc.)

O Professional position (e.g. physician, dentist, lawyer, engineer, etc.)

O Retired

O Unknown / not applicable

Does this parent hold a managerial or executive position?

O Yes

O No

O Unknown

Did you have a part-time job while attending medical school?

O Yes

O No

If yes, are you currently working part-time in addition to attending medical school?

O Yes

O No, because I tried to find a job, but was not able to

O No, because I did not try to find a job

O No, because I quit my job (If so, why did you quit?)

____

Why do you work part-time? (Multiple answers possible)

- ☐ To afford living expenses (rent, groceries, tuition fees)
- ☐ To afford luxury purchases (going out, traveling)
- ☐ To gain work experience (e.g., research experience, etc)
- ☐ Other (please explain) ____

What industries do you work in?

- ☐ Healthcare, hospital
- ☐ Healthcare, other than hospital
- ☐ Science / lab work
- ☐ Office help
- ☐ Retail
- ☐ Restaurant
- ☐ Promotion / animation / event
- ☐ Marketing (online / telephone / offline)
- ☐ Delivery service
- ☐ Private tutoring
- ☐ Babysitting
- ☐ Other (please specify) ____

On average, how many hours do you work per month?

- O <5
- O 5 - 10
- O 10 - 15
- O 15 - 20
- O 20 - 25
- O 25 - 30
- O >30

Has COVID-19 negatively affected your finances due to reduced/cancelled work hours in your part-time job?

- O Yes (please specifiy) ____
- O No

Would you consider taking a loan? (Multiple answers possible)

- ☐ Yes, if government-based
- ☐ Yes, if private-based
- ☐ I already have taken a loan
- ☐ ⊗ No

Do you have any financial support (government-based or private) or savings?

- O Yes
- O No

If yes, what is the nature of your financial support? (Multiple answers possible)

- ☐ Personal savings
- ☐ Parents
- ☐ Other Family or private benefactors
- ☐ Government-based scholarship
- ☐ Government-based loan
- ☐ Private scholarship
- ☐ Private loan from financial institutions
- ☐ Line of credit
- ☐ Other (please specify) ____

If you are being funded by your parents, do you need to pay them back?

O Yes

O No

O Other (please specify (e.g. partial repayment))

____

Do you plan to work in family medicine after graduating from medical school?

O Yes

O I have not decided yet

O No (If not: In which specialty would you like to work after graduating?)

____

Would the repayment of a loan affect your choice for primary care (in favor of a higher payed specialty)?

O Yes

O No

Is there anything else you would like to share regarding the financial impact of COVID-19 on your studies?

____

____

____

Please give us your personal opinion on the current Covid-19 effects on the economy and your individual situation.

	Strongly disagree (1)	Rather disagree (2)	Neutral (3)	Somewhat agree (4)	Strongly agree (5)	Not applicable (6)
1. I anticipate problems with my personal finances due to the Covid-19 recession	O	O	O	O	O	O
2. The fear of losing my part-time job is stressful	O	O	O	O	O	O
3. If I lose my part-time job, my academic performance will be negatively affected	O	O	O	O	O	O
4. Without a part-time job, I will have to take out a loan to stay in medical school	O	O	O	O	O	O
5. Without a part-time job, I will need to drop out of medical school	O	O	O	O	O	O
6. The overall economic situation will worsen within the next months	O	O	O	O	O	O
7. The overall economic situation will worsen within the next years	O	O	O	O	O	O

8. Students from low socioeconomic status (SES) have to work harder to stay in medical school compared to students from a higher SES	O	O	O	O	O	O
9. The loss of part-time job opportunities to afford medical school makes it more of a privilege for students from a higher SES	O	O	O	O	O	O
10. The government should have a backup plan for students affected by the economic regression that allows them to stay in medical school	O	O	O	O	O	O

Attachment 1 to Wohlfarth B, McConnell MM, Huguenin-Dezot M, Nendaz M, Kaderli RM. *Is COVID-19 perceived as a threat to equal career opportunities amongst Swiss medical students? A cross-sectional survey study from Bern and Geneva.* GMS J Med Educ. 2023;40(1):Doc4. DOI: 10.3205/zma001586
